# Detection and Genetic Characterization of Atypical Porcine Pestivirus in Piglets With Congenital Tremors in Southern China

**DOI:** 10.3389/fmicb.2019.01406

**Published:** 2019-06-20

**Authors:** Yongsheng Xie, Xiaoru Wang, Danping Su, Junsen Feng, Liuming Wei, Weiyou Cai, Jinhui Li, Shaorong Lin, He Yan, Dongsheng He

**Affiliations:** ^1^College of Veterinary Medicine, South China Agricultural University, Guangzhou, China; ^2^School of Food Science and Engineering, South China University of Technology, Guangzhou, China

**Keywords:** atypical porcine pestivirus, APPV, congenital tremor, piglets, phylogenetic analysis, genotype 3, Southern China

## Abstract

Atypical porcine pestivirus (APPV) is an RNA virus newly discovered from swine in Asia, Europe, and the Americas. This novel virus has been confirmed as the cause of congenital tremor (CT) in piglets, which causes extensive economic losses to the swine industry. To investigate the genetic diversity and evolutionary relationship of APPV in China, 83 piglet samples with severe CT clinical signs were obtained from 12 commercial swine farms in 3 provinces of Southern China. RT-PCR revealed that the positive rates of APPV were as high as 100% (12/12) for the swine farms and 90.4% (75/83) for the samples. Subsequently, 21 positive samples and 3 positive samples were selected for partial E2 gene and complete polyprotein gene sequencing, respectively. Phylogenetic analysis showed that 62.5% of the sequences belonged to a novel APPV clade provisionally named genotype 3, which showed 81.0–82.1% sequence identity to genotypes 1 and 2. Amino acid sequence alignment showed that E2 protein of genotype 3 has three specific mutation sites, namely I19V, Y82F, and N107G. The results of the present study demonstrate that a novel APPV subgenotype, which is widely distributed in severe CT clinical samples in Southern China, was genetically diverse. We advocate for the inclusion of genotype 3 during revision of the APPV typing method.

## Introduction

Pestiviruses are highly mutable RNA viruses comprising bovine viral diarrhea viruses 1 and 2, border disease virus, and classical swine fever virus (CSFV), which are responsible for huge economic losses in the swine industry worldwide (Postel et al., 2016, 2017; Smith et al., 2017; Zhou et al., 2019). In 2015, Hause et al. (2015) using metagenomic sequencing, found a novel pestivirus, named atypical porcine pestivirus (APPV), in swine serum samples that were positive for porcine reproductive and respiratory syndrome virus. Soon thereafter, this novel virus, which has caused extensive economic losses to the pig industry, was confirmed as the cause of congenital tremor (CT) in piglets in Netherlands, United States, Germany, Austria, China, Spain, Hungary, Brazil, and other countries (Arruda et al., 2016; de Groof et al., 2016; Munoz-Gonzalez et al., 2017; Postel et al., 2017; Schwarz et al., 2017; Yuan et al., 2017; Zhang et al., 2017; Denes et al., 2018; Mosena et al., 2018; Possatti et al., 2018). Recent studies have shown that APPV also circulates in boars in Germany and US; this finding merits further research in this regard (Cagatay et al., 2018; Gatto et al., 2018a).

Pigs affected by CT are commonly known as “shaker pigs” or “dancing pigs” (de Groof et al., 2016; Gatto et al., 2018b; Zhang et al., 2018b; Pan et al., 2019). The main clinical symptoms are rhythmic tremors of the head, abdomen, and limbs. These tremors gradually become more debilitating, causing difficulty in standing or a complete inability to walk, ultimately leading to starvation of affected piglets as they cannot stand to feed (de Groof et al., 2016; Schwarz et al., 2017; Yuan et al., 2017; Gatto et al., 2018b).

APPV is a highly variable single-stranded RNA virus with a genome of approximately 12 kb in length; it contains a long open reading frame (ORF) encoding a polyprotein, composed of four structural proteins (C, Erns, E1, and E2) and eight non-structural proteins (Npro, P7, NS2, NS3, NS4A, NS4B, NS5A, and NS5B) (Hause et al., 2015; Pan et al., 2019). de Groof et al. (2016) provisionally divided APPV into three clusters based on genome analyses. However, as more sequences have been discovered, the phylogenetic tree of APPV has become more complicated (de Groof et al., 2016; Zhang et al., 2018b; Pan et al., 2019). Latest research divided APPV into five different clusters (A-E) (Zhou et al., 2019).

So far, although some novel APPV sequences have been identified (Postel et al., 2017; Zhang et al., 2018b, 2018c; Pan et al., 2019; Zhou et al., 2019), strains available for biological, origin, and evolution analyses are scarce, rendering studies for understanding APPV genetic diversity and evolutionary relationships inadequate. This study aimed to investigate the diversity of APPV sequences and their evolutionary relationships in Southern China to improve understanding of the epidemiological characteristics, evolution, and genetic divergence of APPV.

## Materials and Methods

### Collection of CT Clinical Samples

From May 2017 to March 2018, 83 pathology samples of newborn piglets which died from CT were collected from 12 commercial swine farms in Southern China (Guangdong, Jiangxi, and Anhui provinces), and submitted for immediate diagnostic investigation. Each sample tested included the brain, lung, heart, kidney, spleen, and lymph nodes of the piglet. Seventy samples were from 10 swine farms in Guangdong province, six samples were from a swine farm in Jiangxi province, and seven samples were from a swine farm in Anhui province. Each of the twelve commercial swine farms housed more than 2,000 breeding sows with no pigs being introduced from other countries in the last 2 years. Detailed clinical sample information is shown in Table 1. This work complied with the Laboratory Animals-Guideline of Welfare and Ethics published by the General Administration of Quality Supervision, Inspection, and Quarantine of the People’s Republic of China. Studies were carried out in accordance with animal ethics guidelines and approved by the Animal Care and Use Committee of South China Agriculture University, China.

**Table 1:** Clinical samples collected between 2017 and 2018 in Southern China and results of APPV detection using RT-PCR.

Collection date	Geographical location	Farm ID	Age (days)	Sample type	Clinical signs	Number of samples^x^	Number of APPV
2017.05.31	Anhui	A	1	Tissues^†^	CT^‡^	7	6
2017.06.01	Guangdong	B	1	Tissues	CT	3	3
2017.06.17	Guangdong	C	4	Tissues	CT	7	6
2018.08.10	Guangdong	D	2	Tissues	CT	6	4
2017.09.04	Guangdong	E	1	Tissues	CT	16	16
2017.09.27	Guangdong	F	1	Tissues	CT	5	5
2017.10.14	Guangdong	G	1	Tissues	CT	6	6
2017.11.01	Jiangxi	H	3	Tissues	CT	6	6
2017.11.07	Guangdong	I	5	Tissues	CT	5	4
2017.12.08	Guangdong	J	4	Tissues	CT	5	3
2017.12.13	Guangdong	K	1	Tissues	CT	9	8
2018.03.16	Guangdong	L	3	Tissues	CT	8	8
Total	–	12	–	–	–	83	75 (90.4%)

*^†^Tissues include the brain, lungs, heart, kidneys, spleen, and lymph nodes. ^‡^CT, congenital tremor. ^x^ Each sample was collected from a different piglet.*

### Viral RNA Extraction

Pathology samples were homogenized using TissueLyser II (Qiagen, Hilden, Germany) and diluted 10-fold with 0.1 M phosphate-buffered saline (pH 7.4), frozen and thawed three times, then centrifuged at 8,000 × *g* for 30 min at 4°C. Viral RNA was isolated from the supernatants using the QIAamp Viral RNA Mini Kit (Qiagen, Hilden, Germany) according to the manufacturer’s instructions and immediately stored at -80°C until use.

### Primer Design and RT-PCR Detection of APPV

Based on published APPV sequences, primers specific to conserved regions of the genome (primers F0 and R0; Table 2) were designed for RT-PCR detection of APPV using Primer 6.0 software. RT-PCR amplification was performed using a one-step RT-PCR kit (TaKaRa, Dalian, China) according to the manufacturer’s instructions. The amplification volume was 25 μL, and the amplification conditions were as follows: 50°C for 30 min and 94°C for 2 min for the RT reaction, followed by 35 cycles of amplification at 94°C for 30 s, 56°C for 30 s, and 72 C for 30 s, with a final extension at 72 C for 7 min.

**Table 2:** Primers used for the detection of APPV and amplification of the complete polyprotein gene.

Primer name	Nucleotide sequence (5′ to 3′)	Product size (bp)	Purpose
APPV-4298-F0	CTCACYAGTGATGGGTGGGA	450	Detection and genome sequencing
APPV-4748-R0	CCTATYTTCTTCATGAAYACCATGGC		
APPV-335-F1	GGCGGATGCCTCRGGTAAGA	1937	Genome sequencing
APPV-2271-R1	TGGYTTCACRCATACCCACTGG		
APPV-1837-F2	TTYTTAGACACYATTGGGAGG	788	Genome sequencing
APPV-2624-R2	ACCATWTTTATCTGYTCCATT		
APPV-2250-F3	CCAGTGGGTATGYGTGAARCC	2025	Genome sequencing
APPV-4274-R3	ATAAGRGAGTCATTCTTCTTWGC		
APPV-3380-F4	TTAGGCTTGTCGGAGGTAGTG	1300	Genome sequencing
APPV-4679-R4	TAGCATAAGGCATTGTCGGG		
APPV-4640-F5	TGAAGACTGACCAGAAAGCAC	1393	Genome sequencing
APPV-6032-R5	TCATCTACRTCATARAGACCCC		
APPV-5995-F6	ATAATGAATGAGGACTGGGG	1228	Genome sequencing
APPV-7222-R6	AAGCGACCTGYGTGACAAGAC		
APPV-6860-F7	TAGCATTTGGTGAAAGAGAAYTG	1569	Genome sequencing
APPV-8428-R7	TACCATTGACTAAATAACAGGGG		
APPV-8188-F8	GACAAAATACAYTTYTGGAAAGCAC	1846	Genome sequencing
APPV-10033-R8	CCCACTTGTACATWATTTTGGTGAT		
APPV-9898-F9	ATGCCMAAAAATGAGAAAAGRGA	1569	Genome sequencing
APPV-11466-R9	CTCCATTCATTCAAGTATTTACAACA		

### Amplification and Sequencing of the E2 Gene and Complete APPV Genome

Based on the number of APPV positive samples in each farm (Table 1), we selected 21 positive samples and 3 positive samples from 75 positive samples for partial E2 gene, and complete ORF gene sequencing, respectively. Ten pairs of specific primers (F0–F9 and R0–R9, Table 2) were used to amplify the complete APPV polyprotein gene, of which primers F2 and R2 (Table 2) were used to amplify the E2 gene. The PCR products were purified and then cloned into the pMD19-T vector (TaKaRa, Dalian, China). Ten positive plasmids of each amplicons were selected and sequenced in both directions by Guangzhou BGI. Finally, we obtained 24 APPV sequences (including 3 complete ORF sequences and 21 partial E2 gene sequences) from 12 different swine farms, and these sequences have been deposited in the GenBank database under accession numbers MK347474 to MK347497 (Supplementary Table S2).

### Sequence Analysis

Complete genomic sequences and E2 gene sequences of APPV available in the National Center of Biotechnology Information database (accessed January 2019) were downloaded for phylogenetic and nucleotide alignment analyses, including 30 complete ORF sequences and 46 E2 gene sequences. The MegAlign software was used for multiple sequence alignment analysis. Phylogenetic analysis was performed by the neighbor-joining (NJ) method with 1,000 bootstrap replicates in MEGA 6.06 software.

## Results

### Brief Description of CT at Swine Farms and Detection of APPV by RT-PCR

The clinical samples utilized in this study were all obtained from piglets within the first week of birth. Affected piglets experienced whole-body tremors shortly after birth, leading to difficulty in walking and inability to feed. Many cachexic piglets died within a week of CT onset. The incidence of CT in new-born piglets in different litters varied from 10 to 60%. Piglet mortality rates from CT at swine farms D, E, K, and L (Table 1) averaged at approximately 20%, whereas the mortality rate at the remaining eight farms was significantly lower at 10%.

RT-PCR analysis of the 83 CT samples collected from the 12 commercial swine farms showed that 75 samples (90.4%) were positive for APPV, and swine farm positive rates were as high as 100%, indicating that APPV was directly correlated with CT. In addition, we found APPV infections at a swine farm within Anhui Province of China, which has not been reported before.

### Alignment of Complete Genome and E2 Gene Sequences

Three complete ORF sequences, obtained from two different swine farms in Guangdong province, were named GD-LDCT1, GD-YJHSEY2N, and GD-YJHSEY3N. The ORF sequence alignments revealed that average nucleotide identity (ANI) among these three sequences was 81.2–99.9%, and ANI with reference sequences from China was between 81.0 and 99.6% (Table 3). GD-LDCT1 had the lowest ANI of 81.0% with sequences KY475593 and KY612413 found in Guangdong province by Shen et al. (2018) and Zhang et al. (2017), respectively, whereas GD-YJHSEY2N showed the highest homology at 99.6% to sequence KX950762 found in Guangdong province (Zhang et al., 2017; Table 3). Furthermore, the three complete ORF sequences shared only 81.6–83.7% ANI with complete APPV sequences identified from other countries (Table 3). GD-LDCT1 showed the highest homology of 82.1% with 2 strains (LT594521, KX929062) from Germany and Netherlands and had lowest nucleotide similarity of 81.6% with the strain KU041639 from Germany and strain KU194229 from United States (Arruda et al., 2016; de Groof et al., 2016; Postel et al., 2016; Beer et al., 2017; Table 3). GD-YJHSEY3N shared the highest homology of 83.7% with MF979135 identified in Korea and displayed lowest nucleotide similarity of 83.2% with KU194229 from US (Arruda et al., 2016; Table 3). Taken together, ORF sequence alignment results demonstrated that the three complete ORF sequences possessed low nucleotide identity to reference sequences from other countries but showed higher homology with sequences isolated in China.

**Table 3:** Nucleotide identity (%) of three complete ORF sequences with 30 published reference sequences available in GenBank.

Sequence name	GD-YJHSEY2N	GD-YJHSEY3N	GD-LDCT1	MH493896 China	MH493895 China	MH493894 China	Genotype
GD-YJHSEY2N	–	99.9	81.2	84.2	83.0	80.9	Genotype 2
GD-YJHSEY3N	99.9	–	81.2	84.1	83.0	80.9	
GD-LDCT1	81.2	81.2	–	94.1	96.6	98.7	Genotype 3
MF167290 Germany	83.6	83.6	82.0	82.4	82.1	82.0	Genotype 1
MF167291 Germany	83.6	83.6	82.0	82.4	82.1	81.9	
LT594521 Germany	83.6	83.5	82.1	82.3	82.2	82.1	
MH499643 China	83.3	83.3	81.5	82.0	81.4	81.4	
MH499647 China	83.3	83.3	81.6	82.1	81.5	81.4	
KU194229 United States	83.2	83.2	81.6	82.1	81.6	81.5	
KX929062 Netherlands	83.3	83.3	82.1	82.5	82.0	81.9	
KU041639 Germany	83.6	83.6	81.6	82.0	81.8	81.6	
KX778724 Austria	83.4	83.5	81.9	82.3	82.0	81.7	
KY624591 China	83.4	83.5	81.4	82.1	81.7	81.3	
MF979135 Korea	83.6	83.7	81.7	82.1	81.8	81.3	
MG792803 China	84.4	84.4	81.6	82.1	81.4	81.2	
KY652092 China	83.3	83.3	81.5	82.2	81.5	81.3	
KY475592 China	83.5	83.5	81.5	82.2	81.5	81.3	
MH715893 China	83.5	83.4	81.5	82.1	81.5	81.3	
MH499646 China	83.3	83.3	81.4	82.1	81.4	81.2	
MH499645 China	83.4	83.3	81.5	82.2	81.5	81.3	
KR011347 United States	83.5	83.5	81.7	82.0	81.4	81.5	
MF377344 China	83.1	83.1	81.3	82.0	81.4	81.2	
MH102210 China	83.0	83.0	81.2	81.9	81.2	81.0	
KY475593 China	94.7	94.6	81.0	83.9	82.3	80.8	Genotype 2
MH499642 China	96.2	96.2	81.2	84.1	82.6	80.9	
MH499644 China	96.4	96.4	81.2	84.2	82.6	80.9	
MH499648 China	96.8	96.8	81.1	84.0	82.5	80.8	
KX950761 China	99.5	99.5	81.1	84.0	82.9	80.8	
KX950762 China	99.6	99.5	81.1	84.0	83.0	80.8	
KY612413 China	99.5	99.4	81.0	84.0	82.9	80.8	
MH493896 China	84.2	84.1	94.1	–	95.3	94.1	Genotype 3
MH493895 China	83.0	83.0	96.6	95.3	–	97.5	
MH493894 China	80.9	80.9	98.7	94.1	97.5	–	

**FIGURE 1:**
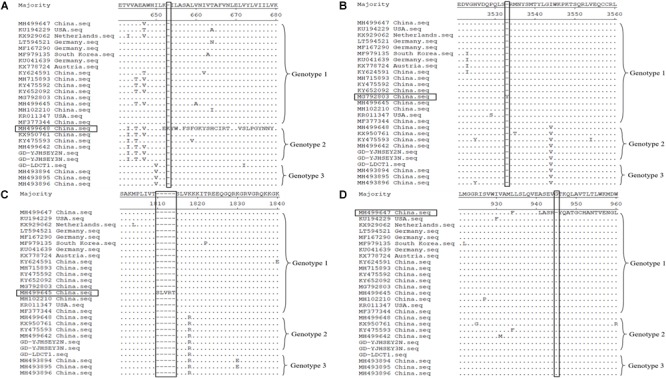
Polyprotein amino acid of APPV alignment by Megalign software available in DNASTAR (http://www.dnastar.com/). Amino acid deletion and insertion regions of the four reference sequences are indicated using rectangular boxes. **(A)** MH499648 (genotype 2) has a lysine inserted at position 653 of the polyprotein. **(B)** MG792803 (genotype 1) has a tyrosine inserted at position 3533. **(C)** MH499645 (genotype 1) has five amino acids (serine, leucine, valine, arginine, and threonine) inserted at position 1810. **(D)** MH499647 (genotype 1) with a deletion of glycine at position 945.

Similar to most published reference sequences, the ORF gene for these three complete ORF sequences had a sequence length of 10,908 bp, without deletions and insertions, with the polyprotein comprising 3,635 amino acids. However, amino acid alignment between the three new APPV sequences and the reference sequences from GenBank showed that a total of four APPV reference sequences had amino acid deletions or insertions in the polyprotein. The ORF of reference sequences MH499648 (genotype 2) and MG792803 (genotype 1, 1c cluster) was 10,911 bp long and translated into 3,636 amino acids (Wu et al., 2018; Zhou et al., 2019). MH499648 has a lysine inserted at position 653 of the polyprotein (Figure 1A), while MG792803 has a tyrosine inserted at position 3533 (Figure 1B). In addition, the sequence MH499645 (genotype 1, 1c cluster) ORF is 10,923 bp long and is translated into 3,640 amino acids (Zhou et al., 2019); it has five amino acids inserted at position 1810 of the polyprotein compared to other APPV sequences (Figure 1C). Furthermore, the MH499647 ORF (genotype 1, 1a cluster) has a length of 10,905 bp and is translated into 3,634 amino acids, with a deletion of glycine at position 945 (Figure 1D; Zhou et al., 2019). Currently, it is unclear whether amino acid deletions or insertions in the polyprotein of APPV can affect the pathogenicity of this virus, and further research is required to determine this.

In addition, partial E2 gene sequence alignments were performed with 46 E2 gene reference sequences. We found that E2 sequences identified in this study shared 79.5–100% ANI with each other (Supplementary Table S1). However, the homology with reference sequences from other countries was between 80.6 and 86.6%, and the homology with reference sequences published in China was 79.3–99.8% (Supplementary Table S1), indicating that the E2 gene sequences in this study showed higher homology to the domestically identified sequences.

### Phylogenetic Analysis and Molecular Features of APPV

**FIGURE 2:**
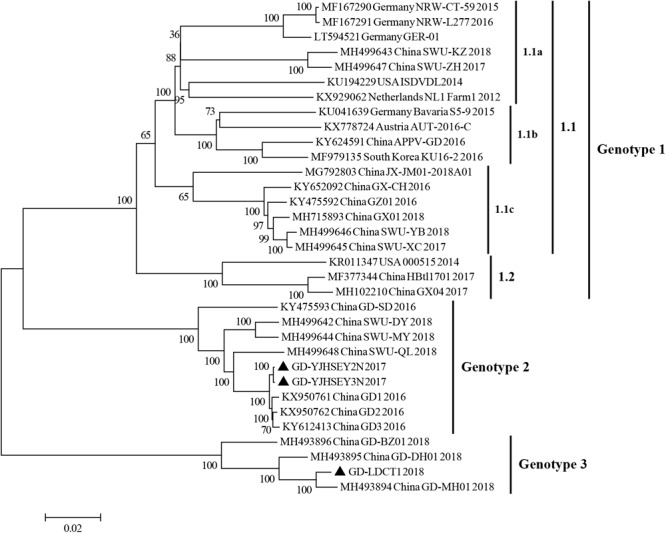
Phylogenetic trees based on the sequences encoding the polyprotein of APPV. Three complete ORF sequences obtained in this study are marked with black triangles. Published sequences, including 30 complete APPV sequences, were downloaded from the GenBank database. MEGA 6.0 software was used to construct the neighbor-joining (NJ) phylogenetic trees using a p-distance method with a bootstrap of 1,000 replicates.

The phylogenetic tree based on complete ORF gene was constructed using the 30 published APPV full-length strains. This analysis showed that all APPV strains from different countries were distributed in different clusters (Figure 2). Strains identified from China were distributed in almost every cluster of the phylogenetic tree, demonstrating that the origin of Chinese APPV strains is very complex. Detailed analysis of the phylogenetic tree showed that all sequences could be divided into three clades. Based on the genotype naming convention for Pestivirus, these three clades were provisionally named genotypes 1, 2, and 3 (Blome et al., 2017; Postel et al., 2018). Genotype 1 included two subclades (1.1 and 1.2) based on evolutionary relationship (Figure 2). In addition, the 1.1 subclade covered most sequences, which can be further divided into three small clusters, 1.1a, 1.1b, and 1.1c; and the only four complete APPV sequences identified in Germany were located in 1.1a and 1.1b clusters (Figure 2). Strains in 1.1c cluster were all isolated from China, and the only complete APPV sequence isolated from Korea belonged to 1.1b (Figure 2). In this study, strains GD-YJHSEY2N and GD-YJHSEY3N belonged to genotype 2, but strains GD-LDCT1 belonged to genotype 3. Unexpectedly, three mutant APPV sequences discovered by Zhang et al. (2018c) also belonged to genotype 3. This is a newly discovered subgenotype with less than 85% ANI compared to genotypes 1 and 2 (Table 3).

**FIGURE 3:**
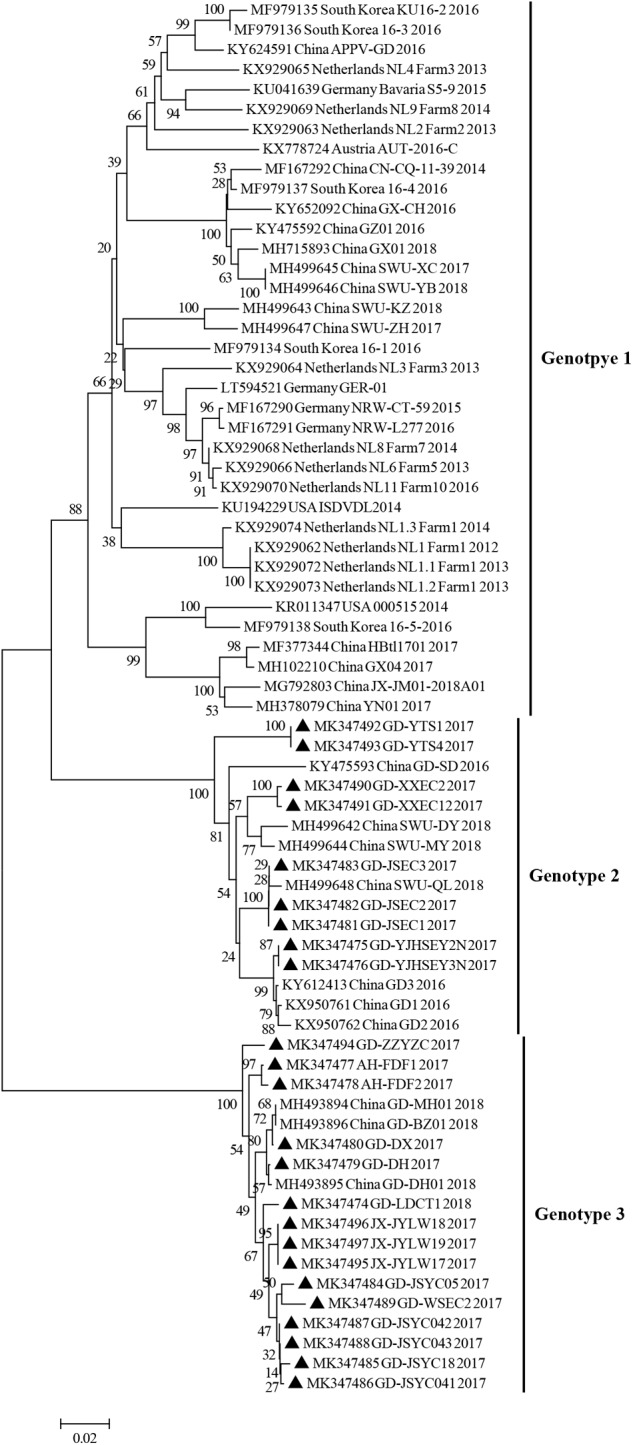
Phylogenetic trees based on the partial sequences (633 bp) of the E2 gene. E2 gene sequences obtained in this study are marked with black triangles. Published sequences, including 46 E2 gene sequences, were downloaded from the GenBank database. MEGA 6.0 software was used to construct the NJ phylogenetic trees using a p-distance method with a bootstrap of 1,000 replicates.

To further explore the evolutionary relationship between E2 sequences identified in this study and previously published APPV E2 sequences, a phylogenetic tree analysis based on partial E2 gene was also performed. Similar to the results obtained using the polyprotein gene phylogenetic tree, all E2 sequences analyzed could be divided into three clades (genotypes 1, 2, and 3) (Figure 3). Further analysis indicated that, in this study, 62.5% of the sequences belonged to genotype 3, 37.5% belonged to genotype 2, and while no sequence belonged to genotype 1.

**FIGURE 4:**
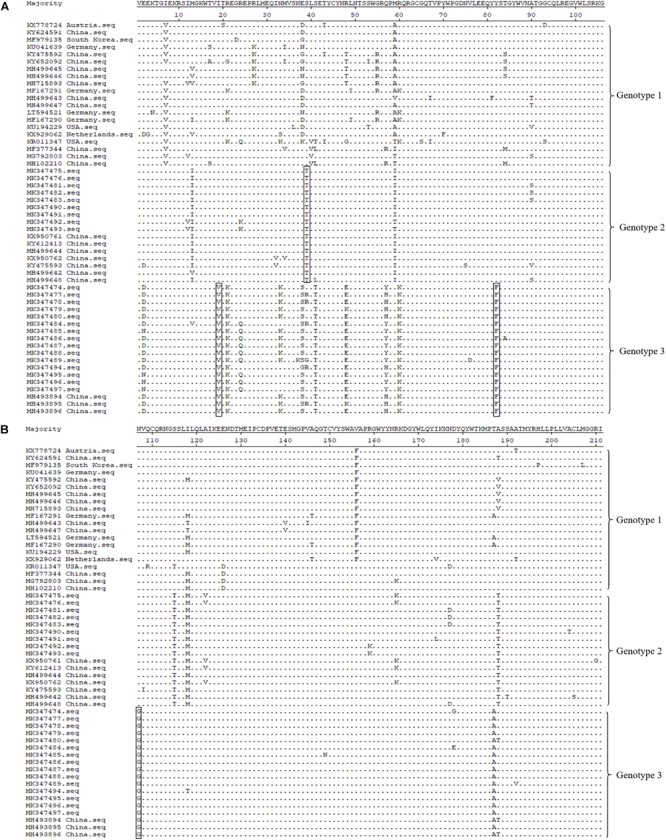
Molecular feature analysis of APPV E2 protein (partial, 211 amino acids). Four specific amino acid mutation sites (I19V, S39T, Y82F, and N107G), which are used for different APPV genotype divisions, are depicted using rectangular boxes. Panel **(A)** displayed is the alignment of the 106 amino acid sequence at the front end of E2 gene. Panel **(B)** displayed is the alignment of the 105 amino acid sequences at the end of E2 gene.

Amino acid alignment of the E2 protein (partial, 211 amino acids) revealed that there were four specific amino acid mutation sites in these three genotypes, which may affect the genotypes of different strains (Figure 4A,B). Genotype 3 contained three amino acid mutations at positions 19, 82, and 107, which were valine, phenylalanine, and glycine, respectively; while genotype 2 contained the S39T mutation; and genotype 1 contained isoleucine, serine, tyrosine, and asparagine at positions 19, 39, 82, and 107, respectively (Figure 4A,B). E2 gene is one of the structural protein genes of APPV and contains several B and T cell epitopes; therefore, E2 protein is subjected to greater immune selection pressure, and we speculated that these mutation sites may be related to some antigenic epitopes of the virus.

## Discussion

Congenital tremor is a common clinical disease in piglets in the swine industry, the clinical symptoms of which were first described nearly a century ago (de Groof et al., 2016; Yuan et al., 2017). After that, studies have shown that factors such as malnutrition, genetics, and viral infections can cause CT clinical signs in piglets (Knox et al., 1978; Vannier et al., 1981; Bradley et al., 1983). In China, the earliest studies on CT can be traced back to the 1960s (Yuan et al., 2017). Through a series of experiments conducted in the following decades, some researchers determined that CT was caused by viral infection, but were not able to find the true pathogen (Yuan et al., 2017). de Groof et al. (2016) confirmed through experiments in pigs that CT was associated with APPV. Then, many studies further confirmed the correlation between APPV and CT. In Austria, APPV infection directly led to a 10% increase in piglet mortality, and APPV antibodies could be detected in both sows and affected piglets; quantitative RT-PCR results showed that adult pigs had a high viral load in the saliva and semen (Schwarz et al., 2017). In Spain, APPV infection can be traced back to 1997, and researchers detected APPV infection in 2-day-old piglets with CT symptoms (Munoz-Gonzalez et al., 2017). In China, Yuan et al. (2017) detected the first APPV sequence in Asia in new-born piglets with CT symptoms, and found the highest viral load in the submaxillary lymph nodes. Two APPV sequences discovered in Guangdong province by Zhang et al. (2017) formed an independent cluster in the phylogenetic tree. Shen et al. (2018) found that APPV infection can cause 60% of new-born piglets to die or be eliminated. In this study, we detected APPV in piglets that died from CT in Southern China; the positive rate of the sample was 90.4% and that of farms was as 100%. Among the piglets infected with CT, the morbidity at different farms ranged from 10 to 60%, and four farms had a mortality rate of 20% because of CT, which resulted in huge economic losses to these farms. Most importantly, two strains of APPV (MK347477 and MK347478) were identified from the Anhui province of China for the first time. This research further confirmed that APPV had a direct correlation with CT, which was consistent with the published studies (Arruda et al., 2016; de Groof et al., 2016; Munoz-Gonzalez et al., 2017; Schwarz et al., 2017; Yuan et al., 2017; Zhang et al., 2017; Shen et al., 2018).

E2 gene is one of the major structural protein genes of APPV. In this study, to analyze the evolutionary relationship of APPV sequences, phylogenetic tree of E2 gene sequences were constructed. Results showed that the phylogenetic trees of the complete ORF genes and E2 genes were similar, and all APPV sequences could be divided into 3 clades (Figure 3, 4). As established, the APPV genome is long and susceptible to mutations, and thus the complete genomic sequence cannot be easily obtained (Postel et al., 2017; Yuan et al., 2017). Therefore, in epidemiological studies, researchers have used different genes (such as E2, NS2-3, NS5B, and Npro) instead of complete genes to construct phylogenetic trees for APPV genotype determination (Beer et al., 2017; Cagatay et al., 2018; Gatto et al., 2018b; Pan et al., 2019; Zhou et al., 2019). However, in this study, the results from phylogenetic analysis of complete ORF genes and E2 genes were quite similar, indicating that the E2 gene could be utilized for phylogenetic analysis in future researches to determine the correct genotypes of different sequences.

It should be noted that many researchers have found sequences of genotype 1 in Chinese swine herds, but sequence alignment and phylogenetic analysis showed that these sequences have high homology and phylogenetic relationship with genotype 1 sequences identified from other countries (Yuan et al., 2017; Zhou et al., 2019). Zhang et al. (2018c) have recently discovered three novel APPV sequences, and ORF sequence alignments revealed that GD-LDCT1 showed the highest ANI of 94.1–98.7% with these three sequences (Table 3); however, the authors did not investigate the prevalence of APPV in China. In this study, phylogenetic analysis showed that these sequences also belonged to genotype 3, and we found many genotype 3 APPV sequences in CT-affected piglets in the southern province of China. Further, our results showed that APPV sequences identified from eight swine farms belonged to genotype 3, indicating a high prevalence of genotype 3. So far, only China has reported sequences of genotype 3 of APPV, and the 12 commercial swine farms in this study had no breeding pigs introduced from other countries in the past 2 years. Sequence alignment results showed that genotype 3 shared less than 85% nucleotide identities with genotypes 1 and 2 (Table 3); thus, this is a novel sequence, and it can be speculated that the genotype 3 APPV strain originated in China rather than being imported from another country.

It is well known that new-born piglets have poor resistance to disease. After APPV infection, CT can cause piglets to walk with difficulty and be unable to feed on colostrum, thereby starving or being crushed to death (Yuan et al., 2017; Gatto et al., 2018b). Vaccines reducing the effects of APPV on new-born piglets may be a highly effective treatment. The E2 protein of CSFV can induce a strong immune response as well as resistance to viral infection; based on this principle, Zhang et al. (2018a) have successfully expressed the E2 protein of APPV *in vitro* in a baculovirus system. Results showed that the E2 protein, combined with a different adjuvant, may induce the production of APPV-specific antibodies in mice, indicating that the E2 protein could potentially be utilized as an effective subunit vaccine to prevent APPV infection (Zhang et al., 2018a).

## Conclusion

This study found APPV in CT samples from 12 commercial pig farms in Southern China. Sequencing and phylogenetic analysis were performed on positive samples. Results clearly showed that 62.5% of the sequences belonged to genotype 3. Thus, further research into modes of transmission and vaccine development is paramount in order to reduce future negative impact to the swine industry.

## Data Availability

The datasets generated for this study can be found in GenBank database, MK 347474 to MK347497.

## Author Contributions

YX conceived and designed the experiments and drafted the manuscript. XW analyzed the data and tools. DS, LW, and WC completed the collection of samples. JF, JL, and SL performed the experiments. HY and DH conceived the study and critically revised the manuscript. All authors read and approved the final version of the manuscript.

## Conflict of Interest Statement

The authors declare that the research was conducted in the absence of any commercial or financial relationships that could be construed as a potential conflict of interest.
