# Supplementary material for: Detection and Genetic Characterization of Atypical Porcine Pestivirus in Piglets With Congenital Tremors in Southern China
Source: Front Microbiol. 2019 Jun 20;10:1406. doi: 10.3389/fmicb.2019.01406 (PMC6596314; doi:10.3389/fmicb.2019.01406)
Supplement: Supplementary file 1 [file Table_1.DOCX]

***Supplementary Material***

**Detection and genetic characterization of atypical porcine pestivirus in piglets with congenital tremors in Southern China**

Yongsheng Xie^1^*, Xiaoru Wang^2^*, Danping Su^1^*, Junsen Feng^1^, Liuming Wei^1^, Weiyou Cai^1^, Jinhui Li^1^, Shaorong Lin^1^, He Yan^2^**, Dongsheng He^1^**

*^1^ College of Veterinary Medicine, South China Agricultural University, Guangzhou, 510642, China.*

*^2^ School of Food Science and Engineering, South China University of Technology, Guangzhou 510641, China.*

Running title: A novel genotype of atypical porcine pestivirus in Southern China

*These authors contributed equally

**Corresponding author:

Dr. He Yan, Ph.D.

Mailing address: School of Food Science and Engineering, South China University of Technology, Guangzhou 510640, China

E-mail Address: yanhe@scut.edu.cn

Tel: +86-20-87113848

Prof. Dongsheng He.

Mailing address: College of Veterinary Medicine, South China Agricultural University, Guangzhou, 510642, China

E-mail Address: dhe@scau.edu.cn

Tel: +86-20-85282537

**Supplementary Table S1.** Nucleotide identity (%) of partial E2 gene sequences identified in this study with 46 published E2 gene reference sequences available in GenBank.

| GenBank number | MK347475 | MK347476 | MK347481 | MK347482 | MK347483 | MK347490 | MK347491 | MK347492 | MK347493 | MK347474 | MK347477 | MK347478 | MK347479 | MK347480 | MK347484 | MK347485 | MK347486 | MK347487 | MK347488 | MK347489 | MK347494 | MK347495 | MK347496 | MK347497 | Genotype |
| --- | --- | --- | --- | --- | --- | --- | --- | --- | --- | --- | --- | --- | --- | --- | --- | --- | --- | --- | --- | --- | --- | --- | --- | --- | --- |
| MK347475 | - | 100 | 97.3 | 97.3 | 97.3 | 96.4 | 96.2 | 94.8 | 94.8 | 81.2 | 81.7 | 81.5 | 81.2 | 81.4 | 80.4 | 80.4 | 80.6 | 80.7 | 80.7 | 79.9 | 81.7 | 81.2 | 81.2 | 81.2 | Genotype 2 |
| MK347476 | 100 | - | 97.3 | 97.3 | 97.3 | 96.4 | 96.2 | 94.8 | 94.8 | 81.2 | 81.7 | 81.5 | 81.2 | 81.4 | 80.4 | 80.4 | 80.6 | 80.7 | 80.7 | 79.9 | 81.7 | 81.2 | 81.2 | 81.2 |  |
| MK347481 | 97.3 | 97.3 | - | 100 | 100 | 96.2 | 96.1 | 94.5 | 94.5 | 81.2 | 81.4 | 81.2 | 81.2 | 81.4 | 80.4 | 80.4 | 80.6 | 80.7 | 80.7 | 79.9 | 81.4 | 81.2 | 81.2 | 81.2 |  |
| MK347482 | 97.3 | 97.3 | 100 | - | 100 | 96.2 | 96.1 | 94.5 | 94.5 | 81.2 | 81.4 | 81.2 | 81.2 | 81.4 | 80.4 | 80.4 | 80.6 | 80.7 | 80.7 | 79.9 | 81.4 | 81.2 | 81.2 | 81.2 |  |
| MK347483 | 97.3 | 97.3 | 100 | 100 | - | 96.2 | 96.1 | 94.5 | 94.5 | 81.2 | 81.4 | 81.2 | 81.2 | 81.4 | 80.4 | 80.4 | 80.6 | 80.7 | 80.7 | 79.9 | 81.4 | 81.2 | 81.2 | 81.2 |  |
| MK347490 | 96.4 | 96.4 | 96.2 | 96.2 | 96.2 | - | 99.5 | 93.8 | 93.8 | 80.9 | 81.4 | 81.2 | 80.9 | 81.0 | 80.1 | 80.1 | 80.3 | 80.4 | 80.4 | 79.6 | 81.4 | 80.9 | 80.9 | 80.9 |  |
| MK347491 | 96.2 | 96.2 | 96.1 | 96.1 | 96.1 | 99.5 | - | 93.7 | 93.7 | 80.7 | 81.2 | 81.0 | 80.7 | 80.9 | 79.9 | 79.9 | 80.1 | 80.3 | 80.3 | 79.5 | 81.2 | 80.7 | 80.7 | 80.7 |  |
| MK347492 | 94.8 | 94.8 | 94.5 | 94.5 | 94.5 | 93.8 | 93.7 | - | 100 | 81.0 | 81.2 | 81.0 | 81.4 | 81.2 | 80.7 | 80.9 | 81.0 | 81.2 | 81.2 | 80.4 | 81.4 | 81.5 | 81.5 | 81.5 |  |
| MK347493 | 94.8 | 94.8 | 94.5 | 94.5 | 94.5 | 93.8 | 93.7 | 100 | - | 81.0 | 81.2 | 81.0 | 81.4 | 81.2 | 80.7 | 80.9 | 81.0 | 81.2 | 81.2 | 80.4 | 81.4 | 81.5 | 81.5 | 81.5 |  |
| MK347474 | 81.2 | 81.2 | 81.2 | 81.2 | 81.2 | 80.9 | 80.7 | 81.0 | 81.0 | - | 97.9 | 97.9 | 98.4 | 98.3 | 98.1 | 98.9 | 99.1 | 99.2 | 99.2 | 98.3 | 97.5 | 98.4 | 98.4 | 98.4 | Genotype 3 |
| MK347477 | 81.7 | 81.7 | 81.4 | 81.4 | 81.4 | 81.4 | 81.2 | 81.2 | 81.2 | 97.9 | - | 99.7 | 98.6 | 98.4 | 97.6 | 98.1 | 98.3 | 98.4 | 98.4 | 97.8 | 98.3 | 97.6 | 97.6 | 97.6 |  |
| MK347478 | 81.5 | 81.5 | 81.2 | 81.2 | 81.2 | 81.2 | 81.0 | 81.0 | 81.0 | 97.9 | 99.7 | - | 98.6 | 98.4 | 97.3 | 98.1 | 98.3 | 98.4 | 98.4 | 97.6 | 97.9 | 97.6 | 97.6 | 97.6 |  |
| MK347479 | 81.2 | 81.2 | 81.2 | 81.2 | 81.2 | 80.9 | 80.7 | 81.4 | 81.4 | 98.4 | 98.6 | 98.6 | - | 99.5 | 97.8 | 98.6 | 98.7 | 98.9 | 98.9 | 97.9 | 98.1 | 98.1 | 98.1 | 98.1 |  |
| MK347480 | 81.4 | 81.4 | 81.4 | 81.4 | 81.4 | 81.0 | 80.9 | 81.2 | 81.2 | 98.3 | 98.4 | 98.4 | 99.5 | - | 97.6 | 98.4 | 98.6 | 98.7 | 98.7 | 97.8 | 97.9 | 97.9 | 97.9 | 97.9 |  |
| MK347484 | 80.4 | 80.4 | 80.4 | 80.4 | 80.4 | 80.1 | 79.9 | 80.7 | 80.7 | 98.1 | 97.6 | 97.3 | 97.8 | 97.6 | - | 98.6 | 98.7 | 98.9 | 98.9 | 98.6 | 97.8 | 98.4 | 98.4 | 98.4 |  |
| MK347485 | 80.4 | 80.4 | 80.4 | 80.4 | 80.4 | 80.1 | 79.9 | 80.9 | 80.9 | 98.9 | 98.1 | 98.1 | 98.6 | 98.4 | 98.6 | - | 99.5 | 99.7 | 99.7 | 98.7 | 97.6 | 99.2 | 99.2 | 99.2 |  |
| MK347486 | 80.6 | 80.6 | 80.6 | 80.6 | 80.6 | 80.3 | 80.1 | 81.0 | 81.0 | 99.1 | 98.3 | 98.3 | 98.7 | 98.6 | 98.7 | 99.5 | - | 99.8 | 99.8 | 98.9 | 97.8 | 99.1 | 99.1 | 99.1 |  |
| MK347487 | 80.7 | 80.7 | 80.7 | 80.7 | 80.7 | 80.4 | 80.3 | 81.2 | 81.2 | 99.2 | 98.4 | 98.4 | 98.9 | 98.7 | 98.9 | 99.7 | 99.8 | - | 100 | 99.1 | 97.9 | 99.2 | 99.2 | 99.2 |  |
| MK347488 | 80.7 | 80.7 | 80.7 | 80.7 | 80.7 | 80.4 | 80.3 | 81.2 | 81.2 | 99.2 | 98.4 | 98.4 | 98.9 | 98.7 | 98.9 | 99.7 | 99.8 | 100 | - | 99.1 | 97.9 | 99.2 | 99.2 | 99.2 |  |
| MK347489 | 79.9 | 79.9 | 79.9 | 79.9 | 79.9 | 79.6 | 79.5 | 80.4 | 80.4 | 98.3 | 97.8 | 97.6 | 97.9 | 97.8 | 98.6 | 98.7 | 98.9 | 99.1 | 99.1 | - | 97.6 | 98.3 | 98.3 | 98.3 |  |
| MK347494 | 81.7 | 81.7 | 81.4 | 81.4 | 81.4 | 81.4 | 81.2 | 81.4 | 81.4 | 97.5 | 98.3 | 97.9 | 98.1 | 97.9 | 97.8 | 97.6 | 97.8 | 97.9 | 97.9 | 97.6 | - | 97.5 | 97.5 | 97.5 |  |
| MK347495 | 81.2 | 81.2 | 81.2 | 81.2 | 81.2 | 80.9 | 80.7 | 81.5 | 81.5 | 98.4 | 97.6 | 97.6 | 98.1 | 97.9 | 98.4 | 99.2 | 99.1 | 99.2 | 99.2 | 98.3 | 97.5 | - | 100 | 100 |  |
| MK347496 | 81.2 | 81.2 | 81.2 | 81.2 | 81.2 | 80.9 | 80.7 | 81.5 | 81.5 | 98.4 | 97.6 | 97.6 | 98.1 | 97.9 | 98.4 | 99.2 | 99.1 | 99.2 | 99.2 | 98.3 | 97.5 | 100 | - | 100 |  |
| MK347497 | 81.2 | 81.2 | 81.2 | 81.2 | 81.2 | 80.9 | 80.7 | 81.5 | 81.5 | 98.4 | 97.6 | 97.6 | 98.1 | 97.9 | 98.4 | 99.2 | 99.1 | 99.2 | 99.2 | 98.3 | 97.5 | 100 | 100 | - |  |
| MF979135 Korea | 84.8 | 84.8 | 84.8 | 84.8 | 84.8 | 84.8 | 84.7 | 84.5 | 84.5 | 81.7 | 81.8 | 81.7 | 81.8 | 81.7 | 81.2 | 81.2 | 81.4 | 81.5 | 81.5 | 80.7 | 81.5 | 81.5 | 81.5 | 81.5 | Genotype 1 |
| MF979136 Korea | 85.5 | 85.5 | 85.5 | 85.5 | 85.5 | 85.5 | 85.3 | 85.2 | 85.2 | 82.3 | 82.5 | 82.3 | 82.5 | 82.3 | 81.8 | 81.8 | 82.0 | 82.1 | 82.1 | 81.4 | 82.1 | 82.1 | 82.1 | 82.1 |  |
| KY624591 China | 85.0 | 85.0 | 85.3 | 85.3 | 85.3 | 84.7 | 84.5 | 85.0 | 85.0 | 81.7 | 82.1 | 82.0 | 82.0 | 82.1 | 81.5 | 81.5 | 81.7 | 81.8 | 81.8 | 81.0 | 81.8 | 81.8 | 81.8 | 81.8 |  |
| KX929065 Netherlands | 84.8 | 84.8 | 85.2 | 85.2 | 85.2 | 85.2 | 85.0 | 84.7 | 84.7 | 82.0 | 82.5 | 82.3 | 82.3 | 82.1 | 82.1 | 82.1 | 82.3 | 82.5 | 82.5 | 81.7 | 82.8 | 82.5 | 82.5 | 82.5 |  |
| KU041639 Germany | 85.0 | 85.0 | 84.8 | 84.8 | 84.8 | 85.5 | 85.3 | 85.3 | 85.3 | 81.7 | 82.1 | 82.0 | 82.0 | 81.8 | 81.8 | 81.5 | 81.7 | 81.8 | 81.8 | 81.4 | 82.1 | 81.8 | 81.8 | 81.8 |  |
| KX929069 Netherlands | 85.6 | 85.6 | 86.1 | 86.1 | 86.1 | 86.1 | 85.9 | 85.3 | 85.3 | 82.5 | 82.9 | 82.8 | 82.8 | 82.3 | 82.3 | 82.3 | 82.5 | 82.6 | 82.6 | 82.0 | 82.9 | 82.6 | 82.6 | 82.6 |  |
| KX929063 Netherlands | 85.2 | 85.2 | 85.8 | 85.8 | 85.8 | 85.2 | 85.0 | 84.7 | 84.7 | 82.9 | 83.6 | 83.3 | 83.3 | 83.1 | 82.9 | 82.8 | 82.9 | 83.1 | 83.1 | 82.5 | 83.6 | 83.1 | 83.1 | 83.1 |  |
| KX778724 Austria | 85.3 | 85.3 | 85.6 | 85.6 | 85.6 | 85.0 | 84.8 | 84.5 | 84.5 | 81.4 | 82.0 | 82.0 | 81.7 | 81.2 | 81.0 | 81.2 | 81.4 | 81.5 | 81.5 | 80.6 | 82.0 | 81.5 | 81.5 | 81.5 |  |
| MF167292 China | 83.3 | 83.3 | 83.6 | 83.6 | 83.6 | 83.3 | 83.3 | 82.8 | 82.8 | 80.9 | 81.7 | 81.5 | 81.4 | 81.2 | 80.7 | 80.7 | 81.0 | 81.0 | 81.0 | 80.6 | 81.4 | 80.7 | 80.7 | 80.7 |  |
| MF979137 Korea | 84.4 | 84.4 | 84.7 | 84.7 | 84.7 | 84.4 | 84.4 | 83.9 | 83.9 | 81.8 | 82.5 | 82.3 | 82.3 | 82.1 | 81.7 | 81.7 | 82.0 | 82.0 | 82.0 | 81.5 | 82.3 | 81.7 | 81.7 | 81.7 |  |
| KY652092 China | 83.1 | 83.1 | 83.4 | 83.4 | 83.4 | 82.8 | 82.8 | 83.1 | 83.1 | 80.9 | 82.0 | 81.8 | 81.4 | 81.2 | 80.9 | 80.7 | 81.0 | 81.0 | 81.0 | 80.6 | 81.2 | 80.9 | 80.9 | 80.9 |  |
| KY475592 China | 83.9 | 83.9 | 84.2 | 84.2 | 84.2 | 83.6 | 83.6 | 83.6 | 83.6 | 81.5 | 82.3 | 82.1 | 82.0 | 81.8 | 81.5 | 81.4 | 81.7 | 81.7 | 81.7 | 81.2 | 82.1 | 81.5 | 81.5 | 81.5 |  |
| MH715893 China | 83.4 | 83.4 | 83.4 | 83.4 | 83.4 | 83.4 | 83.4 | 83.7 | 83.7 | 81.5 | 82.3 | 82.1 | 82.0 | 81.5 | 81.8 | 81.4 | 81.5 | 81.7 | 81.7 | 81.2 | 82.1 | 81.5 | 81.5 | 81.5 |  |
| MH499645 China | 83.7 | 83.7 | 84.0 | 84.0 | 84.0 | 83.7 | 83.7 | 83.4 | 83.4 | 81.5 | 82.3 | 82.1 | 82.0 | 81.8 | 81.8 | 81.4 | 81.7 | 81.7 | 81.7 | 81.2 | 81.8 | 81.5 | 81.5 | 81.5 |  |
| MH499646 China | 83.7 | 83.7 | 84.0 | 84.0 | 84.0 | 83.7 | 83.7 | 83.4 | 83.4 | 81.5 | 82.3 | 82.1 | 82.0 | 81.8 | 81.8 | 81.4 | 81.7 | 81.7 | 81.7 | 81.2 | 81.8 | 81.5 | 81.5 | 81.5 |  |
| MH499643 China | 85.3 | 85.3 | 86.1 | 86.1 | 86.1 | 85.3 | 85.2 | 85.2 | 85.2 | 83.3 | 83.4 | 83.3 | 83.3 | 83.1 | 83.1 | 82.8 | 82.9 | 83.1 | 83.1 | 82.6 | 84.0 | 83.1 | 83.1 | 83.1 |  |
| MH499647 China | 85.0 | 85.0 | 85.6 | 85.6 | 85.6 | 84.7 | 84.5 | 85.2 | 85.2 | 83.1 | 83.6 | 83.4 | 83.4 | 83.3 | 82.9 | 82.6 | 82.8 | 82.9 | 82.9 | 82.5 | 84.2 | 82.6 | 82.6 | 82.6 |  |
| MF979134 Korea | 85.6 | 85.6 | 85.9 | 85.9 | 85.9 | 86.3 | 86.1 | 85.0 | 85.0 | 82.6 | 83.4 | 83.3 | 83.3 | 83.1 | 82.5 | 82.5 | 82.6 | 82.8 | 82.8 | 82.3 | 83.4 | 82.8 | 82.8 | 82.8 |  |
| KX929064 Netherlands | 83.4 | 83.4 | 83.9 | 83.9 | 83.9 | 83.3 | 83.1 | 84.0 | 84.0 | 81.8 | 82.1 | 82.0 | 82.5 | 82.3 | 81.5 | 81.7 | 81.8 | 82.0 | 82.0 | 81.2 | 82.1 | 81.8 | 81.8 | 81.8 |  |
| LT594521 Germany | 84.7 | 84.7 | 85.0 | 85.0 | 85.0 | 85.2 | 85.2 | 84.4 | 84.4 | 82.8 | 83.6 | 83.4 | 83.4 | 83.3 | 82.5 | 82.6 | 82.8 | 82.9 | 82.9 | 82.1 | 83.1 | 82.5 | 82.5 | 82.5 |  |
| MF167290 Germany | 85.2 | 85.2 | 85.3 | 85.3 | 85.3 | 84.8 | 84.8 | 84.7 | 84.7 | 82.3 | 83.4 | 83.3 | 82.9 | 82.8 | 82.0 | 82.1 | 82.3 | 82.5 | 82.5 | 81.7 | 82.9 | 82.3 | 82.3 | 82.3 |  |
| MF167291 Germany | 85.2 | 85.2 | 85.3 | 85.3 | 85.3 | 84.8 | 84.8 | 84.7 | 84.7 | 82.3 | 83.4 | 83.3 | 82.9 | 82.8 | 82.0 | 82.1 | 82.3 | 82.5 | 82.5 | 81.7 | 82.9 | 82.3 | 82.3 | 82.3 |  |
| KX929068 Netherlands | 86.1 | 86.1 | 86.3 | 86.3 | 86.3 | 85.8 | 85.8 | 85.5 | 85.5 | 82.6 | 83.4 | 83.3 | 83.3 | 83.1 | 82.3 | 82.5 | 82.6 | 82.8 | 82.8 | 82.0 | 83.3 | 82.9 | 82.9 | 82.9 |  |
| KX929066 Netherlands | 85.6 | 85.6 | 85.8 | 85.8 | 85.8 | 85.3 | 85.3 | 85.0 | 85.0 | 82.5 | 83.3 | 83.1 | 83.1 | 82.9 | 82.1 | 82.3 | 82.5 | 82.6 | 82.6 | 81.8 | 82.8 | 82.8 | 82.8 | 82.8 |  |
| KX929070 Netherlands | 85.8 | 85.8 | 85.9 | 85.9 | 85.9 | 85.5 | 85.5 | 85.2 | 85.2 | 82.6 | 83.4 | 83.3 | 83.3 | 83.1 | 82.3 | 82.5 | 82.6 | 82.8 | 82.8 | 82.0 | 82.9 | 82.9 | 82.9 | 82.9 |  |
| KU194229 USA | 85.8 | 85.8 | 85.5 | 85.5 | 85.5 | 85.9 | 85.8 | 85.8 | 85.8 | 82.6 | 83.4 | 83.3 | 82.9 | 82.5 | 82.5 | 82.5 | 82.6 | 82.8 | 82.8 | 82.0 | 83.1 | 82.8 | 82.8 | 82.8 |  |
| KX929074 Netherlands | 86.1 | 86.1 | 86.4 | 86.4 | 86.4 | 86.6 | 86.6 | 85.6 | 85.6 | 83.6 | 84.0 | 83.9 | 84.2 | 84.0 | 83.4 | 83.4 | 83.6 | 83.7 | 83.7 | 82.9 | 84.0 | 83.7 | 83.7 | 83.7 |  |
| KX929062 Netherlands | 85.5 | 85.5 | 86.1 | 86.1 | 86.1 | 86.3 | 86.3 | 85.2 | 85.2 | 83.3 | 83.3 | 83.1 | 83.3 | 83.1 | 82.9 | 82.8 | 82.9 | 83.1 | 83.1 | 82.3 | 83.3 | 82.9 | 82.9 | 82.9 |  |
| KX929072 Netherlands | 85.5 | 85.5 | 86.1 | 86.1 | 86.1 | 86.3 | 86.3 | 85.2 | 85.2 | 83.3 | 83.3 | 83.1 | 83.3 | 83.1 | 82.9 | 82.8 | 82.9 | 83.1 | 83.1 | 82.3 | 83.3 | 82.9 | 82.9 | 82.9 |  |
| KX929073 Netherlands | 85.5 | 85.5 | 86.1 | 86.1 | 86.1 | 86.3 | 86.3 | 85.2 | 85.2 | 83.3 | 83.3 | 83.1 | 83.3 | 83.1 | 82.9 | 82.8 | 82.9 | 83.1 | 83.1 | 82.3 | 83.3 | 82.9 | 82.9 | 82.9 |  |
| KR011347 USA | 83.4 | 83.4 | 84.8 | 84.8 | 84.8 | 84.2 | 84.0 | 84.0 | 84.0 | 82.1 | 82.5 | 82.5 | 82.1 | 82.3 | 82.0 | 82.3 | 82.5 | 82.6 | 82.6 | 81.7 | 82.3 | 82.5 | 82.5 | 82.5 |  |
| MF979138 Korea | 84.5 | 84.5 | 85.8 | 85.8 | 85.8 | 84.8 | 84.7 | 84.5 | 84.5 | 82.6 | 82.9 | 82.9 | 82.6 | 82.8 | 82.3 | 82.5 | 82.6 | 82.8 | 82.8 | 81.8 | 83.3 | 82.8 | 82.8 | 82.8 |  |
| MF377344 China | 85.3 | 85.3 | 86.3 | 86.3 | 86.3 | 85.5 | 85.3 | 83.9 | 83.9 | 81.7 | 81.7 | 81.7 | 81.7 | 81.5 | 81.0 | 81.2 | 81.4 | 81.5 | 81.5 | 80.6 | 82.0 | 81.5 | 81.5 | 81.5 |  |
| MH102210 China | 85.2 | 85.2 | 86.1 | 86.1 | 86.1 | 85.0 | 84.8 | 84.0 | 84.0 | 81.8 | 81.8 | 81.8 | 81.8 | 81.7 | 81.2 | 81.4 | 81.5 | 81.7 | 81.7 | 80.7 | 82.1 | 81.7 | 81.7 | 81.7 |  |
| MG792803 China | 85.2 | 85.2 | 86.1 | 86.1 | 86.1 | 85.0 | 84.8 | 83.9 | 83.9 | 81.7 | 81.7 | 81.7 | 81.7 | 81.5 | 81.0 | 81.2 | 81.4 | 81.5 | 81.5 | 80.6 | 82.0 | 81.5 | 81.5 | 81.5 |  |
| MH378079 China | 85.3 | 85.3 | 86.3 | 86.3 | 86.3 | 85.5 | 85.3 | 84.8 | 84.8 | 82.3 | 82.3 | 82.3 | 82.3 | 82.1 | 81.5 | 81.8 | 82.0 | 82.1 | 82.1 | 81.2 | 82.5 | 82.0 | 82.0 | 82.0 |  |
| KY475593 China | 95.4 | 95.4 | 95.3 | 95.3 | 95.3 | 94.3 | 94.2 | 93.7 | 93.7 | 81.0 | 81.4 | 81.2 | 81.0 | 81.2 | 80.6 | 80.6 | 80.7 | 80.9 | 80.9 | 80.1 | 81.5 | 81.4 | 81.4 | 81.4 | Genotype 2 |
| MH499642 China | 96.7 | 96.7 | 96.8 | 96.8 | 96.8 | 96.8 | 96.7 | 93.8 | 93.8 | 80.9 | 81.4 | 81.2 | 80.9 | 81.0 | 80.4 | 80.1 | 80.3 | 80.4 | 80.4 | 79.6 | 81.4 | 80.9 | 80.9 | 80.9 |  |
| MH499644 China | 97.6 | 97.6 | 97.5 | 97.5 | 97.5 | 97.8 | 97.6 | 94.8 | 94.8 | 81.2 | 81.7 | 81.5 | 81.2 | 81.4 | 80.4 | 80.4 | 80.6 | 80.7 | 80.7 | 79.9 | 81.7 | 81.2 | 81.2 | 81.2 |  |
| MH499648 China | 96.8 | 96.8 | 99.5 | 99.5 | 99.5 | 95.9 | 95.7 | 94.3 | 94.3 | 80.9 | 81.0 | 80.9 | 80.9 | 81.0 | 80.1 | 80.1 | 80.3 | 80.4 | 80.4 | 79.6 | 81.0 | 80.9 | 80.9 | 80.9 |  |
| KY612413 China | 99.5 | 99.5 | 97.2 | 97.2 | 97.2 | 96.2 | 96.1 | 94.3 | 94.3 | 80.9 | 81.4 | 81.2 | 80.9 | 81.0 | 80.1 | 80.1 | 80.3 | 80.4 | 80.4 | 79.6 | 81.4 | 80.9 | 80.9 | 80.9 |  |
| KX950761 China | 99.5 | 99.5 | 97.2 | 97.2 | 97.2 | 96.2 | 96.1 | 94.3 | 94.3 | 80.9 | 81.4 | 81.2 | 80.9 | 81.0 | 80.1 | 80.1 | 80.3 | 80.4 | 80.4 | 79.6 | 81.4 | 80.9 | 80.9 | 80.9 |  |
| KX950762 China | 99.2 | 99.2 | 96.8 | 96.8 | 96.8 | 95.9 | 95.7 | 94.0 | 94.0 | 80.6 | 81.0 | 80.9 | 80.6 | 80.7 | 79.8 | 79.8 | 79.9 | 80.1 | 80.1 | 79.3 | 81.0 | 80.6 | 80.6 | 80.6 |  |
| MH493894 China | 81.2 | 81.2 | 81.2 | 81.2 | 81.2 | 80.9 | 80.7 | 81.4 | 81.4 | 98.4 | 98.6 | 98.6 | 99.7 | 99.8 | 97.8 | 98.6 | 98.7 | 98.9 | 98.9 | 97.9 | 98.1 | 98.1 | 98.1 | 98.1 | Genotype 3 |
| MH493896 China | 81.2 | 81.2 | 81.2 | 81.2 | 81.2 | 80.9 | 80.7 | 81.4 | 81.4 | 98.4 | 98.6 | 98.6 | 99.7 | 99.8 | 97.8 | 98.6 | 98.7 | 98.9 | 98.9 | 97.9 | 98.1 | 98.1 | 98.1 | 98.1 |  |
| MH493895 China | 81.2 | 81.2 | 81.2 | 81.2 | 81.2 | 80.9 | 80.7 | 81.4 | 81.4 | 98.3 | 98.7 | 98.4 | 99.8 | 99.4 | 97.9 | 98.4 | 98.6 | 98.7 | 98.7 | 98.1 | 98.3 | 97.9 | 97.9 | 97.9 |  |

**Supplementary Table S2.** List of APPV sequences obtained in this study.

| No. | Strain Name | GenBank accession no. | Province | Farm ID | Gene Type |
| --- | --- | --- | --- | --- | --- |
| 1 | AH-FDF1 | MK347477 | Anhui | Farm A | Partial E2 gene |
| 2 | AH-FDF2 | MK347478 | Anhui | Farm A | Partial E2 gene |
| 3 | GD-DH | MK347479 | Guangdong | Farm B | Partial E2 gene |
| 4 | GD-XXEC2 | MK347490 | Guangdong | Farm C | Partial E2 gene |
| 5 | GD-XXEC12 | MK347491 | Guangdong | Farm C | Partial E2 gene |
| 6 | GD-DX | MK347480 | Guangdong | Farm D | Partial E2 gene |
| 7 | GD-JSYC05 | MK347484 | Guangdong | Farm E | Partial E2 gene |
| 8 | GD-JSYC18 | MK347485 | Guangdong | Farm E | Partial E2 gene |
| 9 | GD-JSYC041 | MK347486 | Guangdong | Farm E | Partial E2 gene |
| 10 | GD-JSYC042 | MK347487 | Guangdong | Farm E | Partial E2 gene |
| 11 | GD-JSYC043 | MK347488 | Guangdong | Farm E | Partial E2 gene |
| 12 | GD-YTS1 | MK347492 | Guangdong | Farm F | Partial E2 gene |
| 13 | GD-YTS4 | MK347493 | Guangdong | Farm F | Partial E2 gene |
| 14 | GD-YJHSEY2N | MK347475 | Guangdong | Farm G | Complete open reading frame |
| 15 | GD-YJHSEY3N | MK347476 | Guangdong | Farm G | Complete open reading frame |
| 16 | JX-JYLW17 | MK347495 | Jiangxi | Farm H | Partial E2 gene |
| 17 | JX-JYLW18 | MK347496 | Jiangxi | Farm H | Partial E2 gene |
| 18 | JX-JYLW19 | MK347497 | Jiangxi | Farm H | Partial E2 gene |
| 29 | GD-WSEC2 | MK347489 | Guangdong | Farm I | Partial E2 gene |
| 20 | GD-ZZYZC | MK347494 | Guangdong | Farm J | Partial E2 gene |
| 21 | GD-JSEC1 | MK347481 | Guangdong | Farm K | Partial E2 gene |
| 22 | GD-JSEC2 | MK347482 | Guangdong | Farm K | Partial E2 gene |
| 23 | GD-JSEC3 | MK347483 | Guangdong | Farm K | Partial E2 gene |
| 24 | GD-LDCT1 | MK347474 | Guangdong | Farm L | Complete open reading frame |
